# Individual differences in face salience and rapid face saccades

**DOI:** 10.1167/jov.24.6.16

**Published:** 2024-06-24

**Authors:** Maximilian Davide Broda, Petra Borovska, Benjamin de Haas

**Affiliations:** 1 Experimental Psychology, Justus Liebig University Giessen, Germany; 2 Center for Mind, Brain and Behavior (CMBB), University of Marburg and Justus Liebig University, Giessen, Germany

**Keywords:** individual differences, salience, rapid saccades, faces

## Abstract

Humans saccade to faces in their periphery faster than to other types of objects. Previous research has highlighted the potential importance of the upper face region in this phenomenon, but it remains unclear whether this is driven by the eye region. Similarly, it remains unclear whether such rapid saccades are exclusive to faces or generalize to other semantically salient stimuli. Furthermore, it is unknown whether individuals differ in their face-specific saccadic reaction times and, if so, whether such differences could be linked to differences in face fixations during free viewing. To explore these open questions, we invited 77 participants to perform a saccadic choice task in which we contrasted faces as well as other salient objects, particularly isolated face features and text, with cars. Additionally, participants freely viewed 700 images of complex natural scenes in a separate session, which allowed us to determine the individual proportion of first fixations falling on faces. For the saccadic choice task, we found advantages for all categories of interest over cars. However, this effect was most pronounced for images of full faces. Full faces also elicited faster saccades compared with eyes, showing that isolated eye regions are not sufficient to elicit face-like responses. Additionally, we found consistent individual differences in saccadic reaction times toward faces that weakly correlated with face salience during free viewing. Our results suggest a link between semantic salience and rapid detection, but underscore the unique status of faces. Further research is needed to resolve the mechanisms underlying rapid face saccades.

## Introduction

Humans rapidly detect whether a face is present in a scene or not (Crouzet, Kirchner, & Thorpe, 2010). This finding is based on the saccadic choice task (SCT), in which participants have to detect a predefined target among two stimuli from different categories as fast and accurately as possible. More precisely, participants fixate centrally until two images from two different categories (a predefined target and a distractor) appear to their left or right, respectively. Their task is to saccade as fast as possible to the target category as soon as the stimuli appear. Previous studies have shown that faces—when being the target category—elicit faster saccadic reaction times (SRTs) than other stimulus categories such as animals or inanimate objects. Humans can reliably detect the presence of a human face within a scene 100 ms after onset (Crouzet et al., 2010). This effect is robust against various stimulus modifications, such as varying eccentricities (Boucart et al., 2016), contrast/orientation inversion (Little, Jenkins, & Susilo, 2021), low-pass filtering (Guyader, Chauvin, Boucart, & Peyrin, 2017), or scrambling face stimuli (Kauffmann, Khazaz, Peyrin, & Guyader, 2021). Because scrambled faces differ in their structure from their intact versions, the programming of rapid face saccades does not seem to rely on holistic face processing and may be driven by particular facial features. The eye region in particular has been suggested to play a role in rapid face detection. Most saccades land near the eyes (Kauffmann et al., 2021) and upper face regions elicit faster SRTs than lower and even whole faces (Broda, Haddad, & de Haas, 2023). This finding is in line with recordings from face-selective cells in macaques showing that faces only containing an eye elicit rapid neural responses (Issa & DiCarlo, 2012). However, it remains unclear whether isolated eye regions are sufficient to elicit rapid saccades in the saccadic choice paradigm.

Trying to predict human gaze behavior has been a focus of vision scientists for decades. Often, research has focused on low-level image properties such as color, orientation, or luminance contrast (Itti, Koch, & Niebur, 1998). However, for complex scenes, low-level models barely perform better than chance (Schütz, Braun, & Gegenfurtner, 2011). Faces, in contrast, reliably attract saccades when present in static or dynamic natural scenes even when observers are not instructed to specifically look at them (Broda & de Haas, 2022a; Broda & de Haas, 2022b; de Haas, Iakovidis, Schwarzkopf, & Gegenfurtner, 2019; Linka & de Haas, 2020; Rider, Coutrot, Pellicano, Dakin, & Mareschal, 2018; Rubo & Gamer, 2018). Generally, humans fixate on faces more often and longer compared with other object categories (de Haas et al., 2019; Linka & de Haas, 2020). Thus, including faces in salience models drastically improves their performance (Cerf, Harel, Einhäuser, & Koch, 2008), as is true for other salient semantic features such as text (Cerf, Paxon Frady, & Koch, 2009; Xu et al., 2014). Faces are salient in the sense that they attract fixations during free viewing. Faces also attract fixation in the SCT. This finding suggests that SRT and performance advantages in the SCT may depend on the same type of salience attracting fixations during free viewing. We previously used the SCT to explore SRTs toward inanimate objects associated with faces, such as face masks or glasses, but found no advantages when compared with cars (Broda et al., 2023). This finding could be the result of the insufficient salience of such objects, which are not known to attract fixations strongly during free viewing. Text, however, is a highly salient semantic category that captures humans’ gaze during free viewing (de Haas et al., 2019; Linka, Sensoy, Karimpur, Schwarzer, & de Haas, 2023; Xu et al., 2014). Nevertheless, it is unknown whether text elements possess a salience advantage in the SCT or if this effect is exclusive to faces.

Even though faces routinely attract saccades during free viewing, observers consistently differ in the degree of this effect for static scenes (Broda & de Haas, 2022a; de Haas et al., 2019; Guy et al., 2019; Linka, Broda, Alsheimer, de Haas, & Ramon, 2022; Peterson & Eckstein, 2013), videos (Broda & de Haas, 2022b; Rubo & Gamer, 2018), or real-world interactions (Guy & Pertzov, 2023; Peterson, Lin, Zaun, & Kanwisher, 2016; Rubo, Huestegge, & Gamer, 2020; Varela, Towler, Kemp, & White, 2023). In static scene viewing, these differences are evident from the first saccade after image onset, showing that individuals consistently differ in early face detection during free viewing (Broda & de Haas, 2022a; Broda & de Haas, 2022b; de Haas et al., 2019; Linka & de Haas, 2020). Interindividual differences in face-directed saccades are stable over time (Broda & de Haas, 2022a; de Haas et al., 2019; Linka & de Haas, 2020) and generalize from static to dynamic scene viewing (Broda & de Haas, 2022b; Peterson et al., 2016). The mechanisms underlying differences in face-directed saccades during free viewing remain unclear, although differences in face-directed saccades have been associated with differences in face recognition (de Haas et al., 2019; Linka et al., 2022), social anxiety (Rubo et al., 2020; Rubo & Gamer, 2018), scene descriptions (Kollenda, Reher, & Haas, 2024), and the experience of urbanicity during childhood (Maran, Hoffmann, & Sachse, 2022).

Recent research from our lab found that saccades targeting faces during free viewing are preceded by shorter fixation durations compared with saccades directed toward inanimate objects (Borovska & de Haas, 2023). This finding suggests the latency advantage for face-directed saccades generalizes between the SRT and natural free viewing, and that at least some mechanisms of face salience may be shared between the SRT and free viewing. The aforementioned individual differences in face salience during free viewing offer an opportunity to test this hypothesis. Individual differences in the proportion of first fixations landing on faces (as discussed elsewhere in this article) seems to be a particularly promising candidate for shared mechanisms with rapid face saccades in the SCT.

Our previous research addressed the question of whether the face advantage in the SCT generalizes to face halves and artificial face-related stimuli (Broda et al., 2023). Here, we investigate whether isolated face features, especially eyes, are sufficient to elicit rapid saccades, similar to whole faces; whether rapid saccades are exclusive to faces or generalize to text; and whether individual differences in early face fixations during free viewing are linked to potential differences for the face advantage in the SCT.

## Methods

### Participants

Seventy-seven healthy adult participants with normal or corrected-to-normal vision took part in the experiment, mean age, 25.23 ± 5.82 years; 53 females. All participants provided written informed consent and the study was approved by the institutional review board and in accord with the declaration of Helsinki. All participants completed both experiments, the SCT and the free viewing task, in separate sessions. All data from the SCT paradigm are new and original. Free viewing data collected during a separate session were used for more studies than the present one (for at least some of our participants). Participants could choose between course credits or 10€/h for reimbursement.

### Apparatus

Participants placed their head in a chin and forehead rest approximately 56 cm from the screen. The experiment was controlled using Psychtoolbox 3.0.16 (Kleiner et al., 2007) in MATLAB R2019a (MathWorks, Natick, MA) on a Windows 10 PC. Gaze data were acquired using an EyeLink 1000 Plus eye tracker (SR Research, Ottawa, Canada) at a frequency of 1 kHz.

### SCT

#### Stimuli

We used 150 grayscale silhouette-cropped car images and 30 silhouette-cropped grayscale images from five categories of interest each: faces, text, noses, mouths, and eyes (Supplementary Figure S1). All images originated from the fLoc functional localizer package (Stigliani, Weiner, & Grill-Spector, 2015). Nose, mouth, and eye stimuli were cropped from the original faces and have been previously used in another experiment (Broda & de Haas, 2023). All stimuli were scaled to the same width of 6.9 degrees visual angle (dva), and placed on a mid-gray background at an eccentricity of 8 dva to the left or right of fixation along the horizontal meridian. We created unique image pairs, each containing an image of a car and an image of a category of interest (i.e., face, text, nose, mouth, or eyes). Images were paired such that the difference in aspect ratios (vertical to horizontal stimulus size) between the members of each pair was minimized.

#### Procedure

In each trial, an image from a category of interest was shown to the left or right of a central fixation point. The corresponding car image appeared on the opposite side. Participants were instructed to saccade as quickly and accurately as possible to the predefined target, which could either be a car or one category of interest, in separate blocks of 60 trials each. The target side was counterbalanced across trials. Each trial started with a central fixation (diameter fixation point: 0.1 dva). After a random interval of 0.8 to 1.6 seconds, the fixation point disappeared and was followed by a gap of 0.2s. Then, a stimulus pair was shown for 0.4 seconds. The next trial started after an interval of 1second. Participants completed sets of 10 trials that were separated by a self-paced drift check which allowed for breaks without leaving the setup. Participants initiated the next set by fixating centrally and pressing the space bar. All categories of interest (i.e., all but cars) served once as target and once as distractor in separate blocks, always paired with cars. In total, participants completed 10 blocks of 6 sets (60 trials) in randomized order. We calibrated participants’ gaze position before each block using a standard nine-point grid. This process allowed for breaks between blocks during which participants could leave the setup. The experiment lasted between 60 and 90 minutes in total.

### Free viewing experiment

#### Stimuli and pixel masks

We used the Object and Semantic Images and Eye-tracking set, which consists of 700 complex natural scenes (Xu et al., 2014). Additionally, we used the head pixel masks for this stimulus set published by Broda and de Haas (2022a) to determine participants’ face salience. Of the 700 images, 454 contained at least 1 face. All scenes were presented at 34.3 × 25.7 dva.

#### Procedure

Participants freely viewed all 700 images for 3 seconds each. Images were presented in the same order for all participants. Participants completed 7 blocks, each containing 100 images. We calibrated participants’ gaze position before each block using a standard nine-point grid. This process allowed for breaks between blocks during which participants could leave the setup. The start of each trial was self-paced and initiated by a central fixation and a button press. The complete experiment lasted between 60 and 90 minutes.

### Data analysis

#### SCT

We included only saccades after the onset of the stimulus pair in each trial and analyzed the first saccade with an amplitude of greater than 1 dva. Trials were excluded if this saccade possessed an initial eccentricity of more than 2 dva, a latency of less than 50 ms, or a duration of more than 100 ms. Only correct responses were considered for SRTs. We excluded two participants with more than 50% invalid trials or incorrect responses. For the remaining participants, an average of 85% of trials were valid and, of those, an average of 73% was correct.

#### Performance

We computed the proportion of correct responses, separately for each participant and block. Performance advantages were always relative to the car category and calculated as the difference in performance when a given category of interest served as target compared with when it served as distractor.

#### SRT

We computed the mean SRT separately for the correct responses of each participant and block. SRT differences were calculated as the difference in SRT of correct responses when a given category of interest served as target compared with when it served as a distractor.

We defined outliers as those instances where individual performance or SRTs deviated by more than 3.5 standard deviations from the group's mean. These outliers were excluded from further analyses. We computed separate repeated-measures analyses of variance (ANOVAs) and subsequent post hoc *t* tests in MATLAB. The family-wise error rate was set to an α of 0.05. We applied the Holm–Bonferroni method to correct for multiple testing (*p* values are reported uncorrected but marked as significant only if they survived correction).

#### Minimum SRTs

We aggregated the SRTs across participants, categorizing them based on response accuracy, separately for each target category. We binned them into 30 bins, each spanning 10 ms, ranging from 60 to 350 ms after stimulus onset. We conducted a binomial test in each bin to determine if the proportion of correct responses significantly exceeded chance level, *p* < 0.05. Minimum SRTs were identified as the earliest significant bin that was followed by at least four consecutive significant bins (cf. Broda et al., 2023; Kauffmann et al., 2021).

#### Face SRT consistency

To test whether the SRT advantage for faces systematically varies across observers, we first applied a stricter exclusion criterion to only include participants with at least 50% valid and correct trials in the block in which whole faces served as the target as well as in the block in which faces served as the distractor category. This led to the exclusion of 17 participants and ensured we only included participants with a sufficient number of trials to allow robust estimates of the individual SRT advantage for whole faces, a crucial prerequisite for analyses pertaining to individual differences (de Haas, 2018). To test whether individuals consistently differed in their SRT differences toward faces, we computed split-half correlations across 1,000 random splits (test distribution). For the null distribution, we used the same splits, but shuffled the data across individuals. We compared the resulting median correlation from the test distribution against the null distribution. The *p* value was determined as the proportion of null distribution events above the median correlation. For explorative reasons, we additionally computed consistencies the same way, including a category specific exclusion criterion (<50% valid/correct trials in the target or distractor block) for text, noses, mouths, and eyes.

#### Free viewing experiment

We only considered participants that were included in the face SRT consistency analysis (as discussed elsewhere in this article). Face fixations were labeled as such if they landed on a head feature in an image or were within a distance of 0.5 dva from the respective feature mask. All fixations earlier than 100 ms after image onset and shorter than 100 ms in duration were excluded from further analyses (cf. Broda & de Haas, 2022a; de Haas et al., 2019).

#### Proportion of first face fixations

We defined the proportion of first face fixations by adding the number of cases in which the first fixation after image onset landed on a face, divided by the number of all first fixations, separately for each individual. We computed split-half correlations across 1,000 random splits (test distribution). For the null distribution, we used the same splits, but shuffled the data across individuals. We compared the resulting median correlation from the test distribution against the null distribution. The *p* value was determined as the proportion of null distribution events above the median correlation. Finally, we correlated face SRT differences with individuals’ proportion of first face fixations. All correlations used Pearson's *r*.

## Results

### Performance

**Figure 1.**
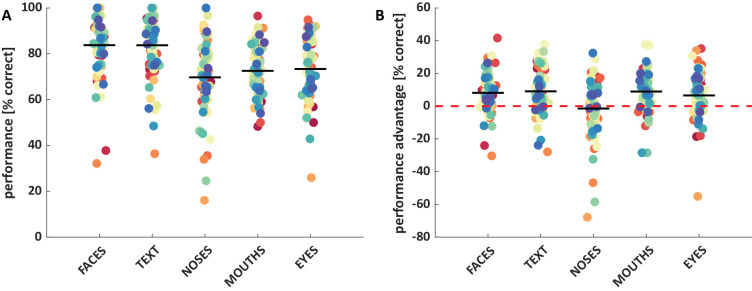
Performance (**A**) and performance advantage (**B**) for each condition. Each dot shows one observer's mean performance (or performance advantage). Black horizontal lines indicate group mean values. Performance corresponds with the proportion of first saccades going to the target in the block in which the respective category served as the target. Performance advantage refers to the difference in the proportion of correct saccadic choices in the block in which the respective category served as target versus blocks in which it served as distractor (and cars served as targets).

The mean proportion of correct responses for saccades toward faces, text, noses, mouths, and eyes were 83.8%, 83.7%, 69.7%, 72.6% and 73.4%, respectively (Figure 1A; Supplementary Figure S2a when they served as distractors). A one-way repeated measures ANOVA revealed a significant main effect of condition, *F*(4, 71) = 42.34, *p* < 0.001, with significantly better performance for face and text than all other categories, all *t* > 6.4, *p* < 0.001, and no further significant differences, but a nonsignificant trend for eyes compared with noses, *t*(71) = 2.20, *p* = 0.031, all other *t* < 1.44, *p* > 0.15 (Supplementary Figure S3a).

The mean performance advantage for saccades toward faces, text, noses, mouths, and eyes (compared with when they served as a distractor) was 8.1%, *t*(73) = 5.84, *p* < 0.001; 9.1%, *t*(73) = 5.86, *p* < 0.001; −1.4%, *t*(73) = −0.70, *p* = 0.484; 8.9%, *t*(74) = 6.34, *p* < 0.001; and 6.6%, *t*(72) = 4.04, *p* < 0.001, respectively (Figure 1B). A one-way repeated measures ANOVA revealed a significant main effect of condition, *F*(4, 71) = 8.27, *p* < 0.001, with a significantly higher advantage for faces, eyes, mouths, and text than for noses, all *t* > 3.4, *p* < 0.001, and no further significant differences, all *t* < 1.1, *p* > 0.29 (Supplementary Figure S3b).

### SRTs

**Figure 2.**
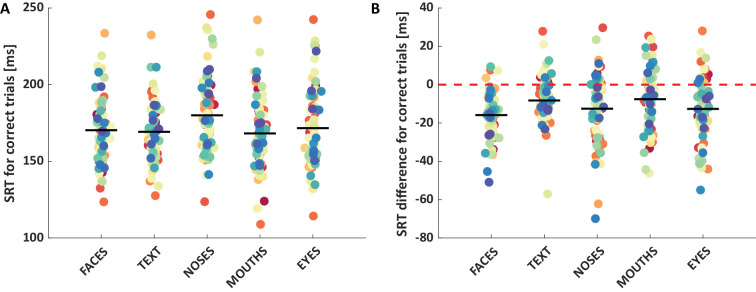
Saccadic reaction time (SRT) (**A**) and SRT advantage (**B**) for each condition. Each dot shows one observer's mean SRT (or SRT advantage). Black horizontal lines indicate group mean values. SRTs correspond with the latency of the first saccade going to the target in correct trials of the block in which the respective category served as the target. SRT differences refer to the difference of SRTs of correct trials in the block in which the respective category served as target versus blocks in which it served as distractor (and cars served as targets).

The mean SRTs of correct responses toward faces, text, noses, mouths, and eyes were 170 ms, 169 ms, 180 ms, 168 ms, and 172 ms, respectively (Figure 2A). A one-way repeated measures ANOVA revealed a significant main effect of condition, *F*(4, 71) = 17.54, *p* < 0.001, with significantly faster SRTs for faces, text, eyes, and mouths than for noses, all *t* < −4.8, *p* < 0.001, and significantly faster SRTs for mouths compared with eyes, *t*(71) = −2.86, *p* = 0.006. There were no further significant differences, a nonsignificant trend for faces vs. mouths, t(71) = −2.20, *p* = 0.031; all other *t* < 1.7, *p* > 0.11 (Supplementary Figure S3c).

The mean SRT difference toward faces, text, noses, mouths, and eyes (compared with when they served as distractors; negative values meaning faster) were −16 ms, *t*(73) = −11.37, *p* < 0.001; −8 ms, *t*(73) = −6.14, *p* < 0.001; −13 ms, *t*(73) = −6.28, *p* < 0.001; −8 ms, *t*(74) = −4.47, *p* < 0.001; and −13 ms, *t*(72) = −7.05, *p* < 0.001, respectively (Figure 2B). A one-way repeated measures ANOVA revealed a significant main effect of condition, *F*(4, 71) = 5.00, *p* < 0.001, with a significantly higher SRT advantage for faces compared with mouths, *t*(71) = −3.95, *p* < 0.001, and text, *t*(71) = −4.44, *p* < 0.001, but no other significant differences, but a nonsignificant trend for eyes compared with text, *t*(71) = −2.19, *p* = 0.032, and mouths, *t*(71) = −2.16, *p* = 0.034; all other *t* < 1.8, *p* > 0.08 (Supplementary Figure S3d).

### Minimum SRTs

**Figure 3.**
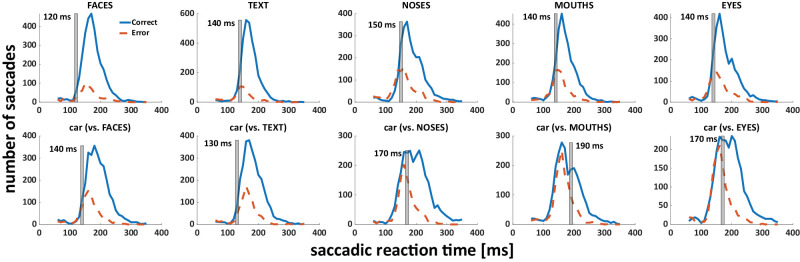
Distribution of saccadic reaction times for correct (blue) and incorrect (dashed red lines) saccades for each condition. The 10-ms bin from which correct responses significantly exceeded incorrect ones is highlighted with a gray bar for each condition. The top row shows conditions in which the respective category of interest was target, and the bottom row the corresponding conditions in which it served as distractor (and cars were targets).

We additionally explored minimum SRTs, determining the lowest SRTs from which the proportion of correct trials became significantly higher than expected by chance. The minimum reaction times for faces, text, noses, mouths, and eyes (vs. cars when the respective category served as distractor) were 120 ms (vs. 140 ms), 140 ms (vs. 130 ms), 150 ms (vs. 170 ms), 140 ms (vs. 190 ms) and 140 ms (vs. 170 ms) respectively (Figure 3).

### Consistencies in SRT differences

**Figure 4.**
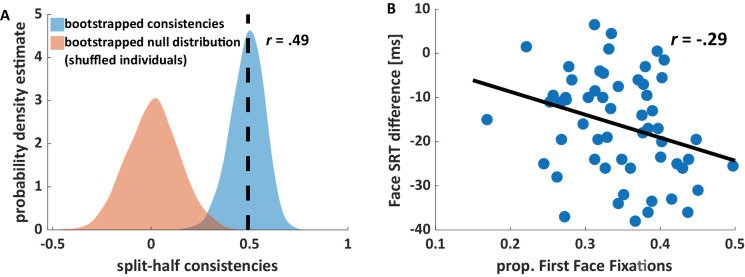
Consistent face saccadic reaction time (SRT) differences. (**A**) Probability density estimate fitted to the histogram of split-half consistencies of face SRT differences based on 1,000 trial-shuffles (blue) and a corresponding null distribution based on shuffling individuals across splits (orange). We found moderately consistent differences between participants (median *r* = 0.49; *p* < 0.001) which is indicated by the dotted line and the median consistency. (**B**) Face SRT differences were significantly correlated with the proportion of first fixations landing on faces during the free viewing task (*r* = −0.29; *p* = 0.026). Negative SRT differences indicated faster reaction times for faces compared with cars. The black line corresponds with the linear least-squares fit.

To test whether participants consistently differed in their face SRT differences, we calculated the median consistency across 1,000 random split-half correlations. Individuals significantly differed in their face SRT differences, *r* = 0.49, *p* < 0.001 (Figure 4A). Exploratory analyses found similar consistencies for text, noses, mouths, and eyes (Supplementary Figure S4).

Next, we tested whether these differences in face SRT differences were correlated with the tendency to fixate faces first when free viewing of complex scenes. Individual differences in the proportion of first fixations landing on faces was highly consistent, *r* = 0.95, *p* < 0.001, replicating previous findings (Broda & de Haas, 2022a; Broda & de Haas, 2022b; de Haas et al., 2019; Guy et al., 2019; Linka et al., 2022; Linka & de Haas, 2020). Crucially, there was a weak but significant correlation between the individual SRT difference for faces and the proportion of first fixations landing on faces during free viewing, *r* = −0.29, *p* = 0.026 (Figure 4B). Participants who showed a higher SRT advantage for faces compared with cars tended to direct more first fixation toward faces during the free viewing task.

## Discussion

Faces elicit faster and more accurate saccades than other types of objects (Crouzet et al., 2010), which may hinge on the eye region (Broda et al., 2023; Kauffmann et al., 2021). However, it remains unclear whether rapid saccades are face specific and whether individual differences in the tendency to immediately fixate faces during free viewing are related to reaction time advantages for faces in the SCT. To tackle these open questions, we contrasted stimuli of whole faces, isolated face regions (eyes, noses, and mouths), and text with images of cars using the SCT. Furthermore, we tested whether face SRT differences varied consistently between individuals and, if so, whether these differences were correlated with general differences in face detection during free viewing.

We found high absolute performances for faces and text as targets (both >83% correct), which was significantly higher than absolute performances for isolated face regions (all <74% correct). However, this effect diminished for performance advantages (i.e., the difference in performance when a category of interest served as a target vs. as a distractor, respectively), because all categories of interest apart from noses showed a significantly stronger performance compared with when they served as distractors and cars served as the target category. These performance advantages over cars may be explained by differences in salience. Faces (e.g., de Haas et al., 2019), face features (e.g., Broda & de Haas 2022a), and text elements (e.g., Xu et al., 2014) have all been shown to attract human gaze when present in a scene. Note, however, that our design followed previous studies in using a singular comparison category (cf. Crouzet et al., 2010; Kauffmann et al., 2019; 2021; Little et al., 2021), leading to far more trials with cars than other types of stimuli. Theoretically, this may cause the observed intercept advantage for non-car stimuli. Crucially, we do not consider this is a concern for our main analyses, namely, testing differences across non-car stimulus categories and across individual participants. Future studies may use more diverse control stimuli and balanced designs (as discussed elsewhere in this article).

Faces and text elicited saccades with similarly low latency. However, although we found a SRT advantage for text over cars, this advantage was significantly lower compared with the advantage for faces over cars, which suggests that faces as distractors are more potent in slowing car-directed saccades than text. This finding is in line with minimum SRTs that showed that participants reliably performed correct saccades earlier for faces than for text. The reaction time advantage for text over cars may point to a general role of semantic salience (i.e., beyond faces) in the rapid detection of objects. Nevertheless, this effect is significantly smaller than that for faces, in line with their unique status for gaze behavior and potential domain-specific mechanisms. The high salience of text elements in scenes (Cerf et al., 2009; de Haas et al., 2019; Xu et al., 2014) rests on the ability to read (Linka et al., 2023), which is in contrast with face salience, which is already present at birth (Fantz, 1963) and possibly even earlier (Reid et al., 2017). This finding suggests that the advantages we found for text over cars may be the result of learned salience and tied to literacy. Alternatively, the text advantage may be mediated by low-level properties (such as contrast levels or spatial frequency spectra) and independent of literacy and acquired semantic salience. Future experiments may juxtapose these hypotheses with the use of well-matched control stimuli and (if possible) by testing pre-literate children in the saccadic choice paradigm.

Noses elicited significantly longer reaction times than all other categories of interest. Similarly, noses were the only category to show no performance advantage and possessed the longest minimum SRTs. However, we found faster reaction times when compared with cars. When freely viewing faces, many observers target the nose region (Broda & de Haas, 2024; Peterson & Eckstein, 2013). The nose has been shown to convey information used to judge a human's age, personality traits, and emotional states (Broda & de Haas, 2023; Diego-Mas, Fuentes-Hurtado, Naranjo, & Alcañiz, 2020). However, information from eyes and mouths is more useful when judging these expressions (Broda & de Haas, 2023). Our findings are in line with these studies, showing that noses are a somewhat salient facial feature, but less so than eyes and mouths.

Whole faces were detected with greater accuracy than all face parts, a greater performance advantage over cars than noses, with a higher reaction time advantage over cars than mouths and text and with the lowest minimum SRT of all stimuli. The previously reported advantages for rapidly detecting the upper face region over other face parts (and even whole faces) (Broda et al., 2023; Kauffmann et al., 2021) did not replicate for the isolated eye region. The previously used upper face stimuli contained the forehead and nose, but we speculated that their advantage was mediated by the eye region alone (Broda et al., 2023). Our current results are not in line with this hypothesis. Instead, the advantage for our previous stimuli may have been mediated by (part of) the typical low-spatial frequency contrast pattern in faces (Dakin & Watt, 2009). The eye region is typically darker compared with the forehead and nose. Previous research has shown that greater contrast results in decreased SRTs (Ludwig, Gilchrist, & McSorley, 2004). Rapid saccades might rely on this contrast between the eye region and its context, which was missing in the current stimuli. Future research could investigate this phenomenon by varying the contrast between isolated eye regions and the background or between the eye region and their surrounding facial regions.

Consistent with previous studies, we placed all stimuli on the horizontal meridian. Previous results show that rapid face-directed saccades toward faces are unaffected by their position in the visual field (Kauffmann et al., 2021; Little et al., 2021). Nonetheless, when freely viewing faces, the eyes are typically located in the upper and the mouth in the lower visual field. Recognition performance decreases when isolated face features are presented at atypical compared with typical visual field locations (de Haas et al., 2016; de Haas et al., 2021; de Haas & Schwarzkopf, 2018). The upper face stimuli used by Broda et al. (2023) included face parts below the eyes, such as the nose or cheeks, so eyes still appeared in participants’ upper visual fields before they executed a saccade, which is in contrast with the current study. Here, the eyes were aligned horizontally with participants' initial gaze position. It is possible that manipulating the visual field position of the isolated eye region would affect performance, and SRTs and upper visual field positions could result in a stronger advantage for the isolated eye region.

We recently found that fixations preceding saccades toward faces in complex natural scenes have a shorter duration than fixations preceding saccades toward inanimate objects (Borovska & de Haas, 2023). This finding suggests that the mechanisms mediating lower SRTs in the saccadic choice paradigm may be at play during free viewing and potentially contribute to face salience in complex scenes. Here, we aimed to test a potential link between face salience in complex scenes and the reaction time advantage for faces in the saccadic choice paradigm using individual differences. We find interindividual differences for the SRT advantage for faces over cars with moderate internal consistency. This result resonates with previous findings regarding individual differences in different aspects of gaze, such as saccade dynamics (Andrews & Coppola, 1999; Bargary et al., 2017; Hancock, Gareze, Findlay, & Andrews, 2012; Rigas, Komogortsev, & Shadmehr, 2016) or semantic salience (Broda & de Haas, 2022a; Broda & de Haas, 2022b; Broda & de Haas, 2024; Guy et al., 2019; Linka et al., 2022; Linka & de Haas, 2020; Peterson et al., 2016; Peterson & Eckstein, 2013; Rubo & Gamer, 2018). We also replicated highly consistent differences in face salience for first fixations during free viewing (cf. Broda & de Haas, 2022a; de Haas et al., 2019). Crucially, we found a weak but significant correlation between interindividual differences in face salience and SRT differences, which could point toward shared mechanisms. This finding is remarkable, given the only moderate consistency of individual estimates of saccadic reaction advantages for faces over cars. Nevertheless, this finding should be considered with caution. We had to exclude more than 20% of our participants owing to a lack of valid data in blocks where faces served as targets or distractors (<30 valid trials). Future studies need to confirm this finding using fewer categories and more trials with whole faces as targets or distractors, which should lead to more precise and robust estimates of individual SRT advantages for faces. Still, this work provides the first evidence that at least some of the variance in SRT differences is shared with face salience during natural free viewing.

In general, the biological mechanisms behind rapid saccades toward faces are still unknown. We replicate reliable detection latencies as low as 120 ms. Macaque studies found reliable face-preferring neural responses in the posterior lateral face patch (Issa & DiCarlo, 2012) and the superior colliculus (Yu, Katz, Quaia, & Messinger, 2023), both with a latency of less than 100 ms. Whether cortical or subcortical structures are involved in triggering rapid saccades toward peripheral faces in humans (Crouzet et al., 2010; Nguyen et al., 2014) remains unclear.

The SCT often uses images of vehicles or buildings as contrast categories. However, we found that both faces (and face parts, except noses) and text elicited superior performance and faster reaction times compared with cars. Previous research has suggested the existence of potential push–pull mechanisms between certain semantic categories. Specifically, face and text fixations (de Haas et al., 2019) and face and body fixations (Broda & de Haas, 2022a; Broda & de Haas, 2022b) are anti-correlated. People who are more prone to fixating faces in natural scenes look less often at text elements or other body parts compared with people who tend to fixate faces less. Here, we have shown consistent individual differences in SRTs toward faces. It would be interesting to see whether similar effects exist in the SCT when contrasting categories with each other, which are anti-correlated in terms of individual semantic preferences during free viewing. Generally, it seems advisable to test a broader range of control stimuli in future experiments to avoid potential specific effects for cars or buildings as control categories.

To conclude, we replicated rapid face saccades using the SCT. The advantage of faces over cars generalized to some extent to isolated face regions and text. However, these advantages were lower when compared with whole faces. We further provide first evidence for consistent individual differences in the SRT advantage for faces, which were weakly but significantly correlated with differences in individual face salience during free viewing. Future research is necessary to test the robustness of this association and potential shared mechanisms between face salience during free viewing and rapid face detection in the saccadic choice paradigm.

## Supplementary Material

Supplement 1

## References

[bib1] Andrews, T. J., & Coppola, D. M. (1999). Idiosyncratic characteristics of saccadic eye movements when viewing different visual environments. *Vision Research,* 39(17), 2947–2953, 10.1016/S0042-6989(99)00019-X. 10492820

[bib2] Bargary, G., Bosten, J. M., Goodbourn, P. T., Lawrance-Owen, A. J., Hogg, R. E., & Mollon, J. D. (2017). Individual differences in human eye movements: An oculomotor signature? *Vision Research,* 141, 157–169, 10.1016/j.visres.2017.03.001. 28373058

[bib3] Borovska, P., & de Haas, B. (2023). Faces in scenes attract rapid saccades. *Journal of Vision,* 23(8), 11–11, 10.1167/JOV.23.8.11. PMC10411644 37552021

[bib4] Boucart, M. V., Lenoble, Q., Quettelart, J., Szaffarczyk, S., Despretz, P., & Thorpe, S. J. (2016). Finding faces, animals, and vehicles in far peripheral vision. *Journal of Vision,* 16(2), 1–13, 10.1167/16.2.10. 27404483

[bib5] Broda, M. D., & de Haas, B. (2022a). Individual differences in looking at persons in scenes. *Journal of Vision,* 22(12), 1–12, 10.1167/jov.22.12.9. PMC9652713 36342691

[bib6] Broda, M. D., & de Haas, B. (2022b). Individual fixation tendencies in person viewing generalize from images to videos. *i-Perception,* 13(6), 1–10, 10.1177/20416695221128844. PMC9638695 36353505

[bib7] Broda, M. D., & de Haas, B. (2023). Reading the mind in the nose. *i-Perception,* 14(2), 1–4, 10.1177/20416695231163449. PMC10028657 36960407

[bib8] Broda, M. D., & de Haas, B. (2024). Individual differences in human gaze behavior generalize from faces to objects. *Proceedings of the National Academy of Sciences of the United States of America,* 121(12), e2322149121, 10.1073/PNAS.2322149121. 38470925 PMC10963009

[bib9] Broda, M. D., Haddad, T., & de Haas, B. (2023). Quick, eyes! Isolated upper face regions but not artificial features elicit rapid saccades. *Journal of Vision,* 23(2), 1–9, 10.1167/JOV.23.2.5. PMC9919614 36749582

[bib10] Cerf, M., Harel, J., Einhäuser, W., & Koch, C. (2008). Predicting human gaze using low-level saliency combined with face detection. *Advances in Neural Information Processing Systems,* 20, 241–248.

[bib11] Cerf, M., Paxon Frady, E., & Koch, C. (2009). Faces and text attract gaze independent of the task: Experimental data and computer model. *Journal of Vision,* 9(12), 1–15, 10.1167/9.12.10. 20053101

[bib12] Crouzet, S. M., Kirchner, H., & Thorpe, S. J. (2010). Fast saccades toward faces: Face detection in just 100 ms. *Journal of Vision,* 10(4), 1–17, 10.1167/10.4.16. 20465335

[bib13] Dakin, S. C., & Watt, R. J. (2009). Biological “bar codes” in human faces. *Journal of Vision,* 9(4), 1–10, 10.1167/9.4.2. 19757911

[bib14] de Haas, B. (2018). How to enhance the power to detect brain–behavior correlations with limited resources. *Frontiers in Human Neuroscience,* 12, 408186, 10.3389/FNHUM.2018.00421/BIBTEX. PMC6198725 30386224

[bib15] de Haas, B., Iakovidis, A. L., Schwarzkopf, D. S., & Gegenfurtner, K. R. (2019). Individual differences in visual salience vary along semantic dimensions. *Proceedings of the National Academy of Sciences of the United States of America,* 116(24), 11687–11692, 10.1073/pnas.1820553116. 31138705 PMC6576124

[bib16] de Haas, B., & Schwarzkopf, D. S. (2018). Feature-location effects in the Thatcher illusion. *Journal of Vision,* 18(4), 1–12, 10.1167/18.4.16. 29710306

[bib17] de Haas, B., Schwarzkopf, S. D., Alvarez, I., Lawson, R. P., Henriksson, L., Kriegeskorte, N., … Rees, G. (2016). Perception and processing of faces in the human brain is tuned to typical feature locations. *Journal of Neuroscience,* 36(36), 9289–9302, 10.1523/JNEUROSCI.4131-14.2016. 27605606 PMC5013182

[bib18] de Haas, B., Sereno, M. I., & Samuel Schwarzkopf, D. (2021). Inferior occipital gyrus is organized along common gradients of spatial and face-part selectivity. *Journal of Neuroscience,* 41(25), 5511–5521, 10.1523/JNEUROSCI.2415-20.2021. 34016715 PMC8221599

[bib19] Diego-Mas, J. A., Fuentes-Hurtado, F., Naranjo, V., & Alcañiz, M. (2020). The influence of each facial feature on how we perceive and interpret human faces. *i-Perception,* 11(5), 1–18, 10.1177/2041669520961123. PMC7533946 33062242

[bib20] Fantz, R. L. (1963). Pattern vision in newborn infants. *Science,* 140(3564), 296–297, 10.1126/SCIENCE.140.3564.296. 17788054

[bib21] Guyader, N., Chauvin, A., Boucart, M., & Peyrin, C. (2017). Do low spatial frequencies explain the extremely fast saccades towards human faces? *Vision Research,* 133, 100–111, 10.1016/J.VISRES.2016.12.019. 28202396

[bib22] Guy, N., Azulay, H., Kardosh, R., Weiss, Y., Hassin, R. R., Israel, S., … Pertzov, Y. (2019). A novel perceptual trait: Gaze predilection for faces during visual exploration. *Scientific Reports,* 9(1), 1–12, 10.1038/s41598-019-47110-x. 31341217 PMC6656722

[bib23] Guy, N., & Pertzov, Y. (2023). The robustness of individual differences in gaze preferences toward faces and eyes across face-to-face experimental designs and its relation to social anxiety. *Journal of Vision,* 23(5), 15–15, 10.1167/JOV.23.5.15. PMC10210514 37212783

[bib24] Hancock, S., Gareze, L., Findlay, J. M., & Andrews, T. J. (2012). Temporal patterns of saccadic eye movements predict individual variation in alternation rate during binocular rivalry. *i-Perception,* 3(1), 88, 10.1068/I0486. 23145268 PMC3485811

[bib25] Issa, E. B., & DiCarlo, J. J. (2012). Precedence of the eye region in neural processing of faces. *Journal of Neuroscience,* 32(47), 16666–16682, 10.1523/JNEUROSCI.2391-12.2012. 23175821 PMC3542390

[bib26] Itti, L., Koch, C., & Niebur, E. (1998). A model of saliency-based visual attention for rapid scene analysis. *IEEE Transactions on Pattern Analysis and Machine Intelligence,* 20(11), 1254–1259, 10.1109/34.730558.

[bib27] Kauffmann, L., Khazaz, S., Peyrin, C., & Guyader, N. (2021). Isolated face features are sufficient to elicit ultra-rapid and involuntary orienting responses toward faces. *Journal of Vision,* 21(2), 1–24, 10.1167/JOV.21.2.4. PMC7873494 33544121

[bib28] Kauffmann, L., Peyrin, C., Chauvin, A., Entzmann, L., Breuil, C., & Guyader, N. (2019). Face perception influences the programming of eye movements. *Scientific Reports,* 9(1), 1–14, 10.1038/s41598-018-36510-0. 30679472 PMC6346063

[bib29a] Kleiner, M., Brainard, D., Pelli, D., Ingling, A., Murray, R., & Broussard, C. (2007). What's new in Psychtoolbox-3? *Perception,* 36, 1–16. ECVP Abstract Supplement.

[bib29] Kollenda, D., Reher, A.-S. V., & Haas, B. de. (2024). Individual gaze predicts individual scene descriptions. *PsyArXiv,* 1–23, 10.31234/OSF.IO/NX7JY. PMC11923161 40108359

[bib30] Linka, M., Broda, M. D., Alsheimer, T., de Haas, B., & Ramon, M. (2022). Characteristic fixation biases in super-recognizers. *Journal of Vision,* 22(8), 1–17, 10.1167/JOV.22.8.17. PMC9344214 35900724

[bib31] Linka, M., & de Haas, B. (2020). OSIEshort: A small stimulus set can reliably estimate individual differences in semantic salience. *Journal of Vision,* 20(9), 1–9, 10.1167/JOV.20.9.13. PMC7509791 32945849

[bib32] Linka, M., Sensoy, Ö., Karimpur, H., Schwarzer, G., & de Haas, B. (2023). Free viewing biases for complex scenes in preschoolers and adults. *Scientific Reports,* 13(1), 1–14, 10.1038/s41598-023-38854-8. 37479760 PMC10362043

[bib33] Little, Z., Jenkins, D., & Susilo, T. (2021). Fast saccades towards faces are robust to orientation inversion and contrast negation. *Vision Research,* 185, 9–16, 10.1016/j.visres.2021.03.009. 33866144

[bib34] Ludwig, C. J. H., Gilchrist, I. D., & McSorley, E. (2004). The influence of spatial frequency and contrast on saccade latencies. *Vision Research,* 44(22), 2597–2604, 10.1016/J.VISRES.2004.05.022. 15358075

[bib35] Maran, T., Hoffmann, A., & Sachse, P. (2022). Early lifetime experience of urban living predicts social attention in real world crowds. *Cognition,* 225, 105099, 10.1016/J.COGNITION.2022.105099. 35334252

[bib36] Nguyen, M. N., Matsumoto, J., Hori, E., Maior, R. S., Tomaz, C., Tran, A. H., … Nishijo, H. (2014). Neuronal responses to face-like and facial stimuli in the monkey superior colliculus. *Frontiers in Behavioral Neuroscience,* 8, 75689, 10.3389/FNBEH.2014.00085/BIBTEX. PMC3955777 24672448

[bib37] Peterson, M. F., & Eckstein, M. P. (2013). Individual differences in eye movements during face identification reflect observer-specific optimal points of fixation. *Psychological Science,* 24(7), 1216–1225, 10.1177/0956797612471684. 23740552 PMC6590077

[bib38] Peterson, M. F., Lin, J., Zaun, I., & Kanwisher, N. (2016). Individual differences in face-looking behavior generalize from the lab to the world. *Journal of Vision,* 16(7), 1–18, 10.1167/16.7.12. 27191940

[bib39] Reid, V. M., Dunn, K., Young, R. J., Amu, J., Donovan, T., & Reissland, N. (2017). The human fetus preferentially engages with face-like visual stimuli. *Current Biology,* 27(12), 1825–1828.e3, 10.1016/J.CUB.2017.05.044. 28602654

[bib40] Rider, A. T., Coutrot, A., Pellicano, E., Dakin, S. C., & Mareschal, I. (2018). Semantic content outweighs low-level saliency in determining children's and adults’ fixation of movies. *Journal of Experimental Child Psychology,* 166, 293–309, 10.1016/J.JECP.2017.09.002. 28972928 PMC5710995

[bib41] Rigas, I., Komogortsev, O., & Shadmehr, R. (2016). Biometric recognition via eye movements: Saccadic vigor and acceleration cues. *ACM Transactions on Applied Perception,* 13(2), 1–21, 10.1145/2842614.

[bib42] Rubo, M., & Gamer, M. (2018). Social content and emotional valence modulate gaze fixations in dynamic scenes. *Scientific Reports,* 8(1), 1–11, 10.1038/s41598-018-22127-w. 29491440 PMC5830578

[bib43] Rubo, M., Huestegge, L., & Gamer, M. (2020). Social anxiety modulates visual exploration in real life – But not in the laboratory. *British Journal of Psychology,* 111(2), 233–245, 10.1111/BJOP.12396. 30945279 PMC7187184

[bib44] Schütz, A. C., Braun, D. I., & Gegenfurtner, K. R. (2011). Eye movements and perception: A selective review. *Journal of Vision,* 11(5), 1–9, 10.1167/11.5.9. 21917784

[bib45] Stigliani, A., Weiner, K. S., & Grill-Spector, K. (2015). Temporal processing capacity in high-level visual cortex is domain specific. *Journal of Neuroscience,* 35(36), 12412–12424, 10.1523/JNEUROSCI.4822-14.2015. 26354910 PMC4563034

[bib46] Varela, V. P. L., Towler, A., Kemp, R. I., & White, D. (2023). Looking at faces in the wild. *Scientific Reports,* 13(1), 1–11, 10.1038/s41598-022-25268-1. 36646709 PMC9842722

[bib47] Xu, J., Jiang, M., Wang, S., Kankanhalli, M. S., & Zhao, Q. (2014). Predicting human gaze beyond pixels. *Journal of Vision,* 14(1), 1–20, 10.1167/14.1.28. 24474825

[bib48] Yu, G., Katz, L. N., Quaia, C., & Messinger, A. (2023). Short-latency preference for faces in the primate superior colliculus. *BioRxiv,* 1–13, 10.1101/2023.09.06.556401. PMC11343682 38959893

