# Supplementary material for: Individual differences in face salience and rapid face saccades
Source: J Vis. 2024 Jun 24;24(6):16. doi: 10.1167/jov.24.6.16 (PMC11204136; doi:10.1167/jov.24.6.16)
Supplement: Supplement 1 [file jovi-24-6-16_s001.docx]

**Supplemental Material**

**
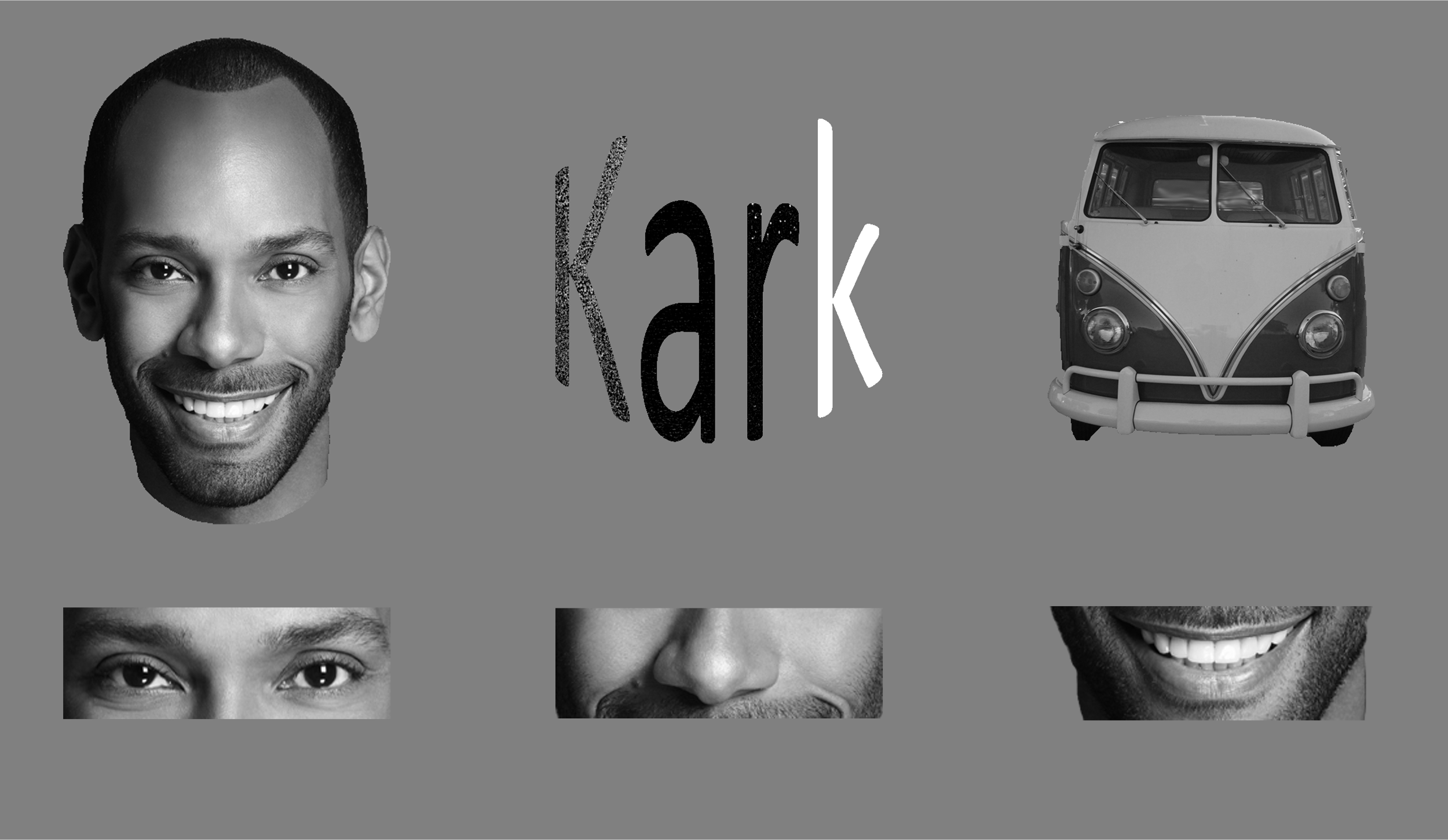
**

Figure S1 Example stimuli for each condition. Faces, text and cars were adapted from the *fLoc functional localizer (Stigliani et al., 2015), and* isolated face regions were manually silhouette-cropped from the faces used in the experiment*. All stimuli were scaled to a width of 6.9 degrees visual angle.*

**
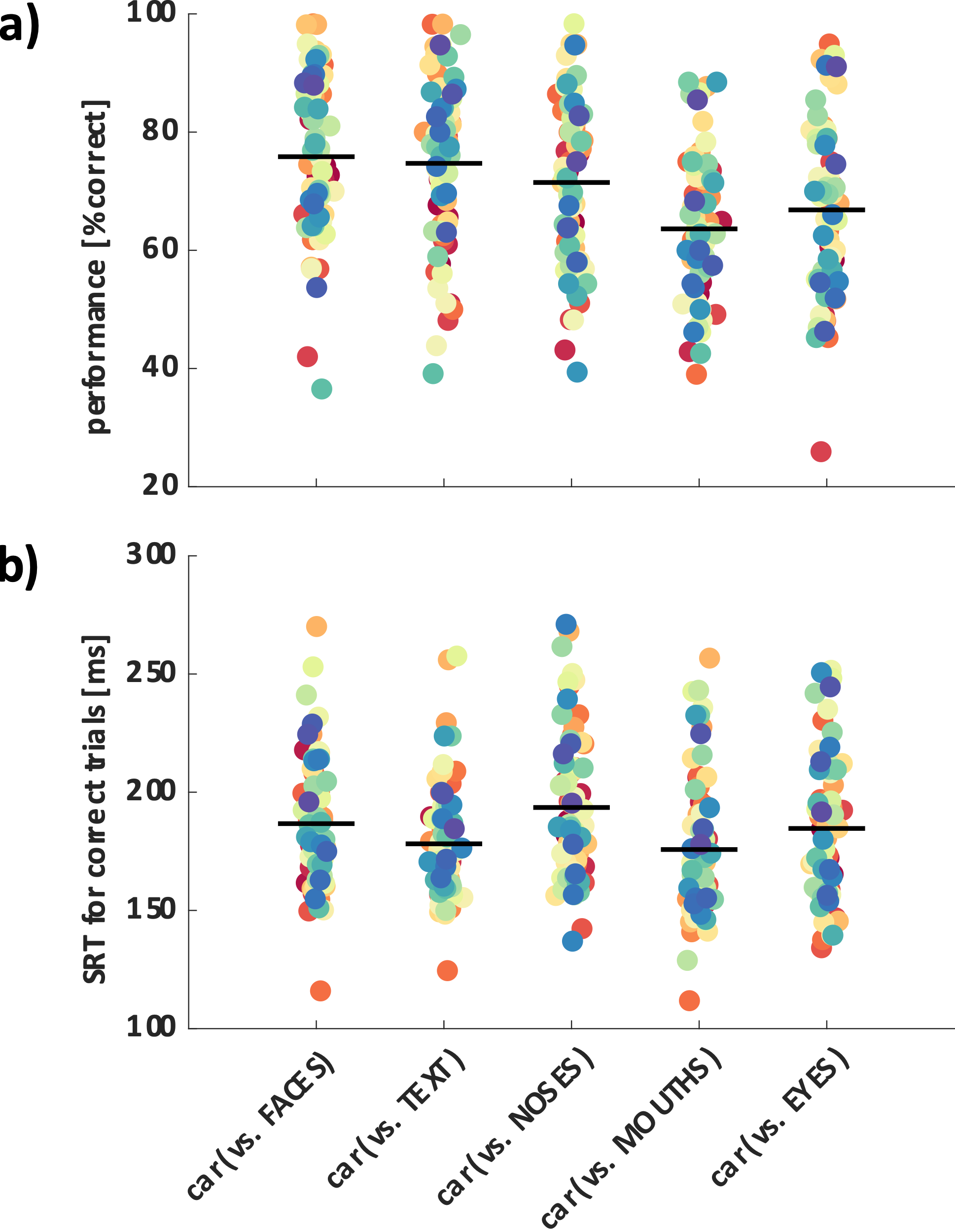
**

Figure S2 Performance (a) and Saccadic reaction time (SRT, b) for each condition when cars served as the target category. Each dot shows one observer's mean performance (or SRT). Black horizontal lines indicate group mean values. Performance corresponds to the proportion of first saccades going to cars in blocks in which cars served as targets. SRTs correspond to saccadic latency towards cars in these correct trials.


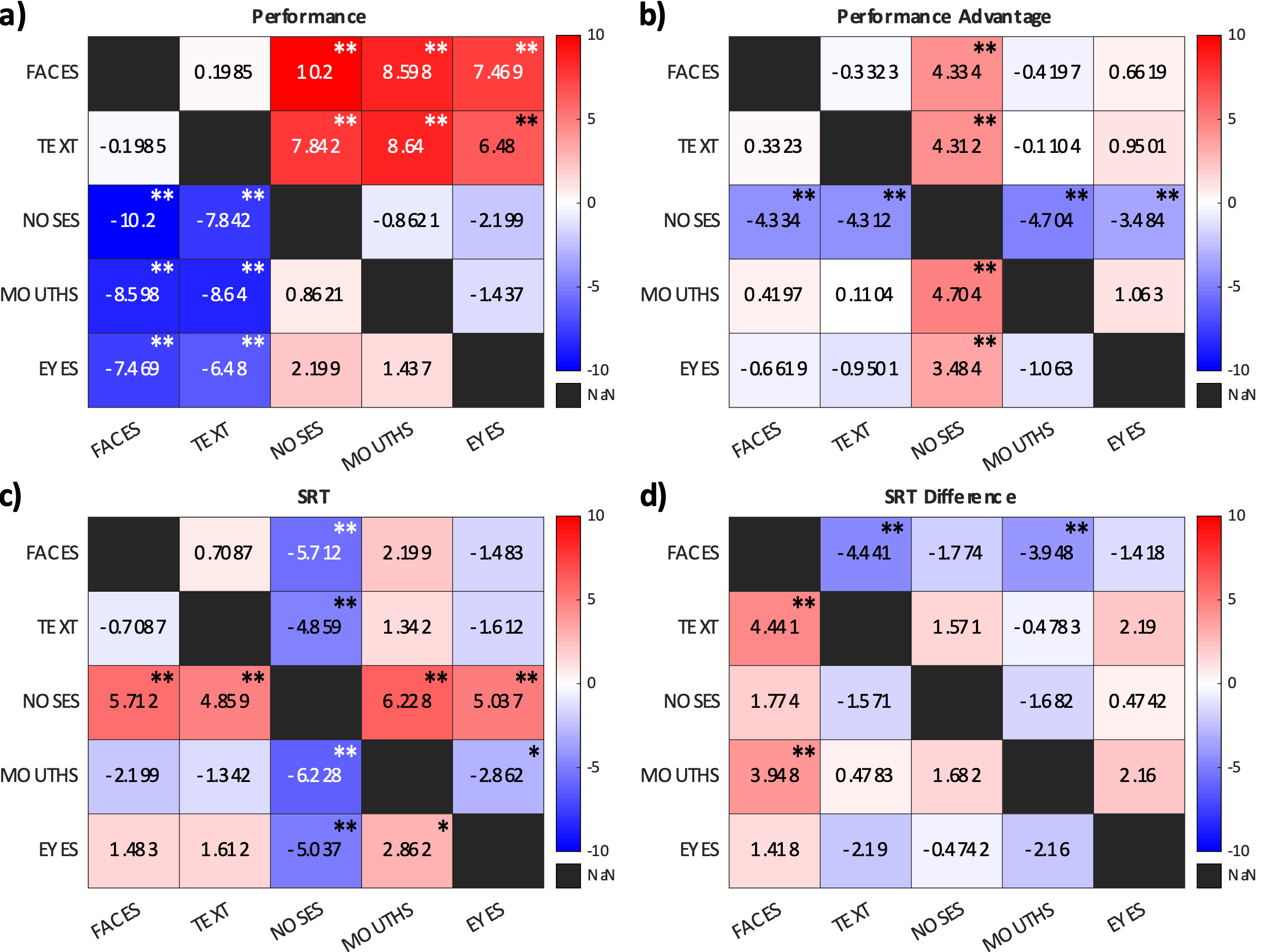


Figure S3 Depiction of all pairwise post-hoc t-tests for (a) performance, (b) performance advantage, (c) SRT, and (d) SRT difference. Negative to positive t values are indicated by color and saturation, as shown on the color bar to the right. Each test compares the respective row vs. column feature. Positive *t*-values indicate a performance advantage or longer reaction times for the respective row vs. column feature. Asterisks indicate statistical significance (Holm-Bonferroni corrected for 10 tests per dependent variable) ** *p* < .001, * *p* < .05.

***
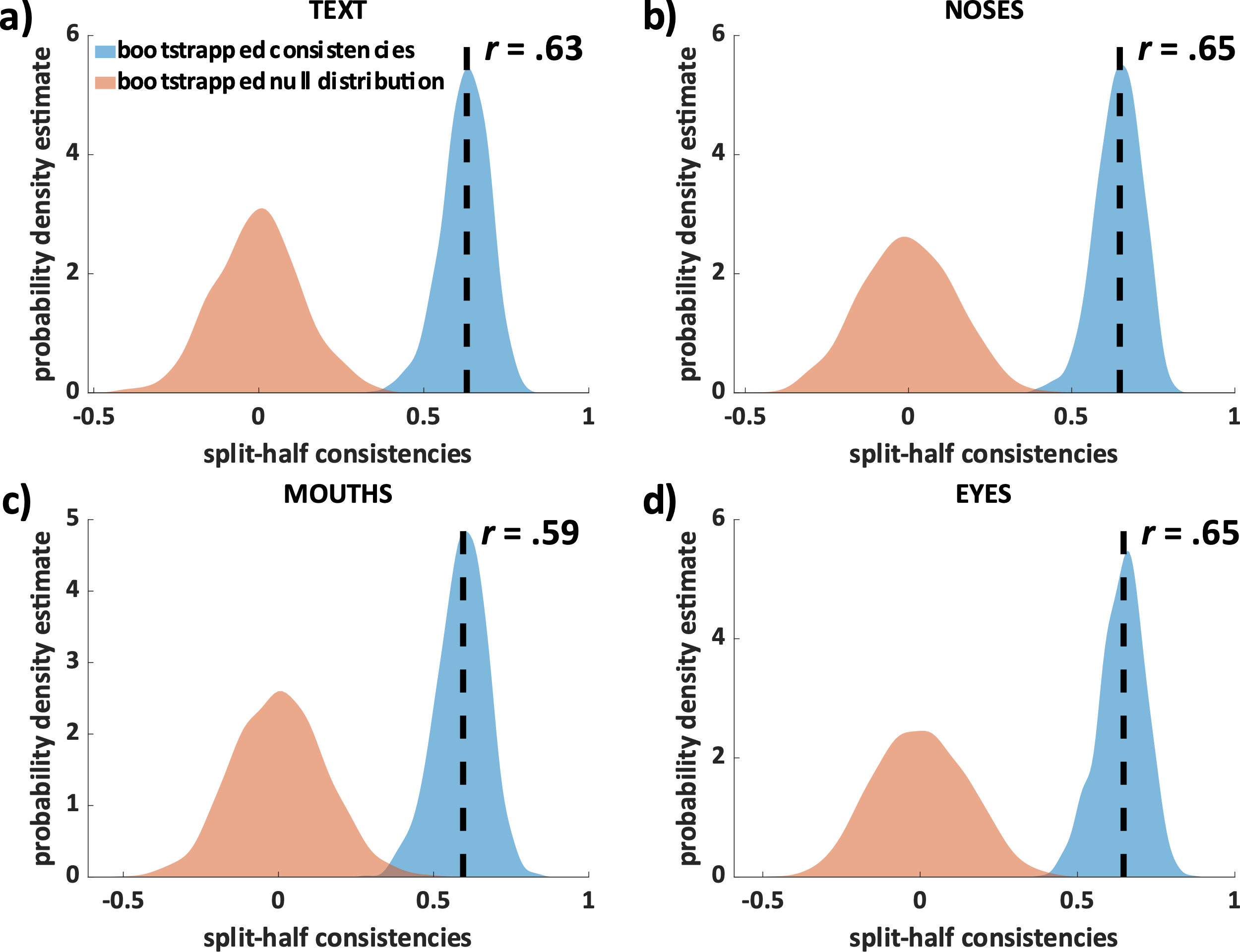
***

Figure S4 Consistent SRT differences.

Depiction of split-half consistencies in SRT differences across 1000 shuffles (blue) and a null distribution shuffled among individuals (orange) for (a) Text, (b) Noses, (c) Mouths, and (d) Eyes. We found moderately consistent differences between participants (median *r* = .59 - .65, all *p* < .001) which are indicated by the dotted line and the median consistency.
